# ESMO 2021—my top three abstracts in breast cancer

**DOI:** 10.1007/s12254-022-00810-z

**Published:** 2022-05-06

**Authors:** Christoph Suppan

**Affiliations:** Clinical Division of Oncology, Department of Internal Medicine, Medical University of Graz, Graz, Austria

**Keywords:** Highlights, Trastuzumab deruxtecan, Pembrolizumab, Ribociclib, CDK4/6 inhibitors

## Abstract

The congress of the European Society of Medical Oncology (ESMO) that recently took place virtually was marked by highlights in many different cancer types. New therapeutic options especially in metastatic breast cancer will hopefully bring a longer life to thousands of patients all over the world. These include new antibody–drug conjugates (ADCs) and checkpoint inhibitors as well as cyclin-dependent kinase (CDK)4/6 inhibitors prolonging overall survival. In Her2-positive advanced breast cancer trastuzumab deruxtecan (T-DXd) compared to trastuzumab emtansine showed a superior benefit in progression-free survival (PFS) in patients who received at least one prior therapy line in the metastatic setting. In the first-line treatment of metastatic triple-negative breast cancer, an overall survival (OS) benefit of pembrolizumab plus chemotherapy versus chemotherapy alone was confirmed for patients with a combined positive score (CPS) ≥ 10. Final results of MONALESSA‑2 demonstrated a great OS benefit for the cyclin dependent kinase (CDK)4/6 inhibitor ribociclib plus endocrine therapy as first-line treatment of patients with hormone receptor (HR)-positive, Her2-negative breast cancer.

## Introduction

Due to the ongoing severe acute respiratory syndrome coronavirus 2 (SARS-CoV-2) pandemic the European Society of Medical Oncology (ESMO) congress 2021 took place virtually again.

In the following short review, I will guide you through my top three abstracts in the field of advanced Her2-positive, triple-negative and hormone receptor (HR)-positive, Her2-negative breast cancer from a clinical point of view.

## Results of the randomized phase III DESTINY-Breast03 study

After the presentation of encouraging results about the activity of trastuzumab deruxtecan (T-DXd) in patients pretreated with trastuzumab emtansine (T-DM1) [1], all of us were waiting for a head-to-head comparison of the two ADCs, i.e., T‑DXd vs T‑DM1 in patients with Her2+ metastatic breast cancer. DESTINY-Breast03 is an open label, multicenter, phase III trial of 524 patients with unresectable or metastatic Her2-positive breast cancer who had previously received trastuzumab and a taxane in the advanced or metastatic setting [2]. They were randomized 1:1 to receive T‑DXd 5.4 mg/kg (*n* = 261) or T‑DM1 3.6 mg/kg every 3 weeks (*n* = 263). The primary endpoint was progression-free survival (PFS); key secondary endpoint was overall survival (OS). About half of the patients received one prior therapy line in the metastatic setting, the rest two or more lines. All of the patients were trastuzumab-pretreated, about 60% also received pertuzumab. About 20% of the patients in this trial had a history of brain metastases. The median age of the patients in the T‑DXd arm was 54.3 years (range 27.9–83.1 years). Median follow-up in the T‑DXd arm was 16.2 months and 15.3 months in the T‑DM1 arm.

The PFS was not reached in the T‑DXd arm and was 6.8 months in the T‑DM1 arm, for an impressing hazard ratio (HR) of 0.28 (95% confidence interval [CI] 0.22–0.37, *p* = 7.8 × 10^−22^). The OS did not reach prespecified cut-off for significance (HR 0.56, 95% CI 0.36–0.86, *p* = 0.007) which was not very surprising given the short follow-up. The confirmed objective response rate (ORR) was 79.7% under T‑DXd versus 34.2% under T‑DM1 (*p* < 0.0001). 16.1% of the patients treated with T‑DXd had a complete response and 63.6% a partial response. The disease control rate (DCR) was 96.6% for T‑DXd and 76.8% for T‑DM1. After a median treatment duration of 14.3 months with T‑DXd and 6.9 months with T‑DM1, treatment-related events occurred in 98.1% and 86.6%. The most common treatment-related adverse event which led to discontinuation of T‑DXd was interstitial lung disease (ILD)/pneumonitis. In 2 of 27 cases grade 3 ILD was diagnosed, there were no grade 4 or grade 5 cases. Serious treatment-related adverse events were seen in 10.9% in the T‑DXd arm versus 6.9% in the T‑DM1 arm. The most common treatment-related adverse events leading to dose reduction of T‑DXd were nausea and neutropenia; in case of T‑DM1 thrombocytopenia and increase of AST/ALT.

Due to this important trial, the ESMO guidelines have already implemented T‑DXd as a treatment option in case of metastatic Her2-positive breast cancer progressing after first-line treatment with a taxane plus trastuzumab/pertuzumab [3]. Assuming that 30–50% of patients with advanced Her2-positive breast cancer will develop brain metastases, this is a subgroup of special interest which is also considered in the current guidelines [4]. There is an urgent need of developing new treatment options which are also effective in the brain. Now, together with the tyrosine kinase inhibitor tucatinib, which was examined in combination with trastuzumab and capecitabine in pretreated patients [5], there seem to be two new promising treatment alternatives for second- or third-line setting.

## KEYNOTE-355

In this trial, the final results of a randomized, double-blind, phase 3 study of first-line pembrolizumab + chemotherapy versus placebo + chemotherapy for metastatic triple-negative breast cancer were presented. A total of 847 patients with metastatic triple-negative breast cancer who did not receive prior treatment in the metastatic setting were randomized 2:1 to pembrolizumab and chemotherapy versus placebo and chemotherapy [6]. Chemotherapy backbone was nab-paclitaxel, paclitaxel or carboplatin/gemcitabine. The coprimary endpoints were PFS and OS, stratified by PD-L1 expression (combined positive score [CPS] ≥ 10 or ≥ 1) and in the intent-to-treat population. Secondary endpoints included ORR, DCR and safety.

In all, 75.1% in both arms were PD-L1 positive with a CPS ≥ 1. 38.9% in the pembrolizumab arm had a CPS ≥ 10. 54.9% received carboplatin/gemcitabine, while 54.1% in the pembrolizumab arm were treated with a taxane. Around one third of the patients had de novo metastasis, while about 20% of all patients had a disease-free interval less than 12 months. The median age of the patients in the pembrolizumab arm was 53 years (range 25–85 years). The median follow-up was 44 months. The OS for patients with a CPS ≥ 10 was 23 months in the pembrolizumab arm versus 16.1 months in the placebo arm (95% CI 0.55–0.95, HR 0.73). The PFS was prolonged from 5.6 to 9.7 months (95% CI 0.50–0.88, HR 0.66). The ORR was 52.7% versus 40.8% with a DCR of 65% when treated with pembrolizumab. The duration of response was 12.8 months.

The OS for patients with a CPS ≥ 1 was 17.6 months when treated with pembrolizumab and 16 months in the placebo arm which was not statistically significant (95% CI 0.72–1.04, HR 0.86). The median PFS was prolonged from 5.6 to 7.6 months (95% CI 0.62–0.91, HR 0.75). A similar trend was seen in the ITT population when patients reached an OS of 17.2 with pembrolizumab versus 15.5 months with placebo (95% CI 0.76–1.05, HR 0.89). Treatment-related adverse events of any grade occurred in 96.3% in the pembrolizumab group and in 95% in the placebo group, with 68% and 67% who experienced grade 3 to grade 5 events. Immune-mediated adverse events occurred in 26.5% under pembrolizumab and in 6.4% under placebo. These adverse events included hypothyroidism, hyperthyroidism, pneumonitis, colitis, or severe skin reactions. With these positive results, pembrolizumab is now the second available checkpoint inhibitor in Europe for first-line treatment of patients with metastatic triple-negative breast cancer and a CPS ≥ 10. One important advantage of pembrolizumab in this setting is that it can be combined with three different chemotherapy regimens, while atezolizumab is only approved together with nab-paclitaxel [7].

## Overall survival results from the phase 3 MONALEESA-2 trial

For this trial, 668 postmenopausal patients with metastatic HR positive (HR+), Her2-negative advanced breast cancer treated with endocrine therapy ± ribociclib were randomized 1:1 to receive either the cyclin dependent kinase (CDK)4/6 inhibitor ribociclib 600 mg per day 3 weeks on/1 week off, or placebo together with the aromatase inhibitor letrozole [8]. Patients did not receive a prior therapy for advanced disease. The primary endpoint was PFS, key secondary endpoint OS. The median duration of follow-up was 80 months. The addition of ribociclib to letrozole resulted in a median OS of 63.9 months versus 51.4 months for placebo (95% CI 0.63–0.93, HR 0.76, *p* = 0.004). The OS benefits emerged at about 20 months and increased over time. At 6 years, the OS rate was 44.2% for ribociclib versus 32% for placebo. As expected, the benefit of ribociclib was seen across all key subgroups. Among patients who discontinued the study treatment, 87.8% of the patients in the ribociclib arm received a subsequent therapy. About one third were then treated with hormone therapy alone, about 17% received chemotherapy. However, the start of the first chemotherapy was prolonged from 38.9 months in the placebo arm to 50.6 months with ribociclib (95% CI 0.61–0.91, HR 0.74). Most events occurred in the first 12 months of treatment. Rates of grade 3/4 adverse events in the ribociclib and placebo arms were neutropenia (63.8 and 1.2%), hepatobiliary toxicity (14.4 and 4.8%), prolonged QT interval (4.5 and 2.1%) and interstitial lung disease/pneumonitis (0.6 and 0%). The median age of the patients in the ribociclib arm was 62 years (range 23–91 years).

After this long follow-up the role of a CDK4/6 inhibitor plus hormone therapy as a first-line treatment for patients with metastatic HR-positive, Her2-negative breast cancer is obvious. Until now, the three available agents abemaciclib, palbociclib and ribociclib seem to be equally effective in the metastatic setting, whereas ribociclib is the only one that showed a consistent OS benefit regardless of endocrine therapy partner or therapy line.

### Take home message

Metastatic breast cancer is a heterogenous disease that requires different targeted therapies: the above discussed abstracts that were presented at the virtual ESMO congress in 2021, again reflect the complexity of the treatment landscape as well as the different patient outcomes due to tumor biology. The new ADC trastuzumab deruxtecan as well as the checkpoint inhibitor pembrolizumab are the new kids on the block in advanced breast cancer and true gamechanger in at least two breast cancer subtypes. In the meantime, agents such as CDK4/6 inhibitors are becoming outdated and provide us with an increasing OS benefit underlining the importance of longer follow-up.
